# Fibroblast Growth Factor Receptor-Mediated Activation of AKT-β-Catenin-CBP Pathway Regulates Survival and Proliferation of Murine Hepatoblasts and Hepatic Tumor Initiating Stem Cells

**DOI:** 10.1371/journal.pone.0050401

**Published:** 2012-11-30

**Authors:** Nirmala Mavila, David James, Sarah Utley, Nguyen Cu, Orly Coblens, Katrina Mak, C. Bart Rountree, Michael Kahn, Kasper S. Wang

**Affiliations:** 1 Saban Research Institute, Children’s Hospital Los Angeles, Los Angeles, California, United States of America; 2 Department of Biochemistry and Molecular Biology and Norris Comprehensive Cancer Center, Keck School of Medicine, University of Southern California, Los Angeles, California, United States of America; 3 Keck School of Medicine, University of Southern California, Los Angeles, California, United States of America; 4 Pediatric Gastroenterology, Bon Secours St. Mary’s Hospital, Richmond, Virginia, United States of America; Children‚s Hospital Los Angeles, United States of America

## Abstract

Fibroblast Growth Factor (FGF)-10 promotes the proliferation and survival of murine hepatoblasts during early stages of hepatogenesis through a Wnt-β-catenin dependent pathway. To determine the mechanism by which this occurs, we expanded primary culture of hepatoblasts enriched for progenitor markers CD133 and CD49f from embryonic day (E) 12.5 fetal liver and an established tumor initiating stem cell line from *Mat1a^−/−^* livers in media conditioned with recombinant (r) FGF10 or rFGF7. FGF Receptor (R) activation resulted in the downstream activation of MAPK, PI3K-AKT, and β-catenin pathways, as well as cellular proliferation. Additionally, increased levels of nuclear β-catenin phosphorylated at Serine-552 in cultured primary hepatoblasts, *Mat1a^−/−^* cells, and also in *ex vivo* embryonic liver explants indicate AKT-dependent activation of β-catenin downstream of FGFR activation; conversely, the addition of AKT inhibitor Ly294002 completely abrogated β-catenin activation. FGFR activation-induced cell proliferation and survival were also inhibited by the compound ICG-001, a small molecule inhibitor of β-catenin-CREB Binding Protein (CBP) in hepatoblasts, further indicating a CBP-dependent regulatory mechanism of β-catenin activity.

*Conclusion:* FGF signaling regulates the proliferation and survival of embryonic and transformed progenitor cells in part through AKT-mediated activation of β-catenin and downstream interaction with the transcriptional co-activator CBP.

## Introduction

Despite progress in characterizing the physiology of hepatic progenitor/stem cells, numerous aspects of signaling pathways regulating these progenitor/stem cells remain undefined. During embryogenesis, hepatic bud formation is induced from the foregut endoderm via instructive Fibroblast Growth Factor (FGF) signaling from adjacent mesenchyme of the developing heart and septum transversum [1]–[3]. At different stages of fetal and postnatal life, the population of progenitor cells, termed hepatoblasts, which likely represent a heterogeneous mixture of precursor cells, variably express genes specific to hepatocytes (*Albumin*, *Hnf4α*), biliary epithelial cells (*Cytokeratin-19*) and putative stem/progenitor cell markers (*Epcam*, *Cd133* and *Cd49f*) [4]–[9]. Under circumstances where liver regeneration via hepatocyte proliferation is impaired, there is evidence of FGF signaling activation promoting the proliferation of epithelial progenitor cells in response to liver injury [10]. Further elucidation of these pathways could provide insight not only into repair mechanisms involving progenitor cells, but also into the biology of liver tumors such as hepatoblastoma, which are theorized to arise from clonal expansion of malignantly transformed progenitor/stem cells [11], [12].

The Fibroblast Growth Factor (FGF) family comprises 22 known ligands, which bind promiscuously and with variable specificity to four tyrosine kinase-type FGF Receptors (R), encoded by genes with multiple alternative splicing loci [13], [14]. Notably FGF7 and FGF10, two key ligands involved in epithelial organogenesis and repair, exhibit greatest affinity for the FGFR2IIIb isoform over other FGFR [15], [16]. FGFR activation is known to regulate the downstream mitogen-activated protein kinase (MAPK) and phospho-inositol-3-kinase (PI3K) pathways [13], [14]. Calmont *et al* demonstrated that during the initial stages of hepatogenesis, the FGF-mediated MAPK pathway regulates endodermal cell specification with induction of hepatic genes, such as *Albumin*, whereas PI3K pathway activation promotes liver growth during early embryonic liver development [17].

The β-catenin pathway is an evolutionarily well-conserved signaling pathway essential to normal cellular processes such as proliferation, survival, and differentiation; it is also involved in self-renewal of progenitor cell populations during embryogenesis, tissue regeneration and repair [18]–[23]. The canonical pathway involves binding of the Wingless/Integrated (Wnt) ligand to the Frizzled receptor. Via a series of intermediate steps, β-catenin is activated and released from the Glycogen Synthase Kinase-3β (GSK3β)/Axin/Adenomatous Polyposis Coli (APC) complex prior to entry into the nucleus. Within the nucleus, β-catenin binds to TCF/LEF transcription factors and regulates expression of various downstream genes. Direct phosphorylation of β-catenin at Serine-552 by AKT leads to expression of a series of anti-apoptotic and cell survival genes [24], [25].

β-catenin activity is also regulated through interaction with two nuclear transcriptional co-activators: Cyclic AMP responsive element binding protein (CBP) and its highly related paralog p300. Both CBP and p300 are transcriptional co-activator proteins that can acetylate histones and non-histone proteins such as β-catenin [26]–[29]. Both proteins regulate gene transcription through protein-protein interactions with transcription factors and chromatin-remodeling complexes [30]. β-catenin interacts with CBP to promote survival and proliferation of embryonic and somatic stem cells, whereas its interaction with p300 promotes the initiation of differentiation of stem cells [31], [32]. CBP is also required for self-renewal of hematopoietic stem cells, while p300 regulates hematopoietic differentiation [33], [34]. Additionally, TCF/β-catenin/CBP-mediated transcription decreases the rate of neuronal progenitor cell differentiation, whereas β-catenin interaction with p300 induces differentiation [35].

During early hepatogenesis around E10.5, *Fgf10* is expressed by mesenchymal cells within the adjacent septum transversum and thereafter, by fetal hepatic stellate cells. FGF10 induces downstream activation of β-catenin in hepatoblasts likely through FGFR2IIIb activation [36]. However, the mechanism by which FGFR2IIIb activates β-catenin and regulates the survival of hepatic progenitor/stem cells is not clear.

In the present study, we utilize *in vitro* culture techniques of whole mount embryonic liver, primary hepatoblasts and tumor initiating liver stem cells to characterize in greater detail the link between FGFR signaling, β-catenin activation, and progenitor cell proliferation.

## Materials and Methods

### Animal Use

C57BL6 wild-type (Jackson laboratories, Harbor, ME) mice were bred, maintained at The Saban Research Institute (TSRI) animal care facility, and handled in accordance with IACUC rules and regulations of the TSRI at Children’s Hospital Los Angeles.

### Cell Culture

The *Mat1a^−/−^* cell line, originally described by Rountree *et al* [37], was grown on standard tissue culture plates (BD Biosciences) in DMEM (Invitrogen) containing 10% Fetal Bovine Serum (FBS, Invitrogen), 100 units/100 µg/ml Penicillin/Streptomycin (Gibco, Carlsbad, CA), 1 µg/ml Insulin (Sigma-Aldrich, St. Louis, MO), 10^−7^ M Dexamethasone (Sigma-Aldrich), and 10 mM Nicotinamide (Sigma-Aldrich) ± 250 ng/ml Fungizone (Gibco, added only for primary culture) in a cell culture incubator maintained at 37°C and 5% CO_2_. Cells were trypsinized using 0.05% Trypsin-EDTA (Gibco, Carlsbard, CA) and passaged every 3–4 days.

For serum starvation, cells were washed with sterile phosphate buffered saline (PBS) twice and media was changed to DMEM containing Dexamethasone and Nicotinamide minus serum or insulin for 16 hours. FGF signaling was stimulated as described previously by growing cells in human rFGF7 or rFGF10 (R &D Systems) conditioned media at 250 ng/ml for 1 hour unless otherwise described [36]. For loss-of-function analyses, we utilized ICG-001, a small molecule inhibitor that specifically disrupts β-catenin’s interaction with CBP [38] and Ly294002, a PI3K-AKT inhibitor, at the well-established dose of 10 µM concentration for the indicated time in each experiment.

### Immunofluorescence Staining

Embryonic livers were isolated from E12.5 C57BL6 wild-type mouse embryos, fixed in 4% paraformaldehyde (PFA, Poly Sciences Inc., Warrington, PA) and embedded in paraffin blocks. 5 µm thick sections were used for immunofluorescence staining. De-paraffinized and antigen-retrieved tissue sections were incubated with respective primary antibodies for 16 hours at 4°C after blocking with 5% goat serum at room temperature for 10 minutes. For detection, we used secondary antibodies conjugated with Cy3, Cy5, or FITC incubated at room temperature for 1 hour. For immunocytochemistry, cells were fixed in 4% PFA for 30 minutes and washed in PBS for 5 minutes twice. The cells were permeabilized with Tris-buffered saline-Triton X-100 (0.5%) for 10 minutes and then washed in PBS for 5 minutes twice. Non-specific binding was reduced by blocking with 5% goat serum (Sigma-Aldrich) for 45 minutes at room temperature. The cells were then incubated with primary antibody for 16 hours at 4°C (see Table S1). Signals were detected by adding secondary antibody conjugated either with goat-anti-mouse Cy3/Cy5/FITC, goat anti-rat Cy3, or goat anti-rabbit Cy3/Cy5 (1∶200, Jackson Immuno Research Laboratories, West Grove, PA). Fluorescence images were acquired using Leica DM5500B immunofluorescence microscope using Leica Suite Advanced Fluorescence (LAS AF) 6000 software (Wetzlar, Germany). Phase contrast pictures were acquired using Evos Advanced Microscopy Group (AMG) transmitted light microscope coupled with Evos *xl* software (AMG, Bothell, Washington, USA).

### Isolation of Embryonic Hepatoblasts

Embryonic liver progenitor cells were isolated from E12.5 embryos of C57BL6 mice. Isolated livers were digested with 0.03% collagenase (Sigma-Aldrich) in DMEM at 37°C for 30 minutes after which enzymatic activity was stopped using 10% FBS. Digested liver cell suspensions were then passed through 70 µm sterile filters (BD Biosciences, Franklin Lakes, NJ) to obtain single cell suspensions. Cells were spun down at 500×g for 5 minutes at 4°C. Cell depletion or selection was performed using the Milteni immuno-magnetic beads (Milteni Biotech Inc., Auburn, CA) conjugated with respective antibodies according to the manufacture’s protocol and protocols published previously [37]. Briefly, cells were washed with ice-cold 1× Magnetic Assisted Cell Separation (MACS) buffer (PBS containing 0.5% BSA, 10 mM CaCl_2_, and 2 mM EDTA). CD45^pos (positive)^ cells were depleted and CD133^pos^ or CD49f^pos^ cells were selected according to manufacturer protocol.

Cell viability was assessed by 0.4% Trypan blue (Gibco) staining. Approximately 1×10^5^ or 2.5×10^5^ viable cells were plated on Laminin-Poly-lysine coated culture slides or 3.5 cm Laminin coated culture plates (BD Biosciences). Media was changed the following day and every other day thereafter as described above. All studies on primary cultured embryonic hepatoblasts were carried out between 3–6 days after plating.

### Purification of RNA and Gene Expression Analysis

2×10^5^ cells were plated on 6-cm tissue culture plates. Cells were serum starved the following day for 16 hours in 0% FBS ± rFGF7/10 conditioned media. Total RNA was isolated by Trizol reagent (Invitrogen, Carlsbad, CA) according to manufacturer’s instructions. RNA purity was assessed by the 260/280 and 260/230 nm absorbance ratio of 2 or greater. 1 µg of total RNA was used for cDNA synthesis using the Bio-Rad iScript cDNA synthesis kit (Bio-Rad Life Science, Hercules, CA). Analysis of gene expression via Reverse-Transcription Polymerase Chain Reaction (RTPCR) was performed on Bio-Rad C1000 Thermal cycler using Taq PCR Master Mix Kit (Qiagen, Valencia, CA) and intron spanning gene specific primers (Table S2). Quantitative Real-Time PCR (qPCR) was performed using cDNA using Light-Cycler Taqman Master (Roche Applied Science, Indianapolis, IN) and probes from the Universal Probe Library (Roche Applied Science) using intron spanning, gene specific primers. Relative expression levels were calculated by Δ–Δ C_t_ method. *Actin* was used to normalize the gene expression.

### Cell Proliferation and BrdU Labeling Assay

3×10^4^ *Mat1a^−/−^* cells were plated on an eight well culture slide (BD Biosciences) and next day serum starved for 16 hours. Cells were then treated with rFGF7/10 (0–250 ng/mL culture media) for 48 hours, and total cell number was counted using a hemocytometer. For BrdU (5-bromo-2′-deoxyuridine) incorporation, 24 hours after plating, cells were serum starved for 16 hours ± CBP inhibitor, ICG-001, or PI3K-AKT inhibitor, Ly294002, and treated with rFGF7/10 and BrdU for 3 hours. In the case of embryonic HPC, 1×10^5^ MACS-sorted CD133^pos^CD49f^pos^ CD45^neg (negative)^ cells were cultured for 3 days in eight-well Laminin-poly-lysine coated culture slides. Cells were then serum starved (0.5% serum, no insulin) for 6 hours in ± ICG-001 and treated with rFGF10/7 and BrdU for 3 hours. The cells were fixed in 4% PFA for 30 minutes at room temperature, 2 N HCl for 30 minutes, and then washed twice in PBS. Cells were blocked with 5% goat serum for 45 minutes at room temperature and incubated with anti-BrdU antibody for 16 hours at 4°C. The signals were detected with Cy3 conjugated anti-mouse secondary antibody. BrdU positive cells were counted in 4–5 20× high power field images using NIH Image J software and compared with control.

### Western Blot Analysis

2×10^5^ *Mat1a^−/−^* cells were plated on 6-cm tissue culture plate and serum starved for 16 hours. FGF signaling was activated as described above. Total protein lysates were prepared from cells using Radio Immuno Precipitation Assay (RIPA) buffer (50 mM Tris-HCl, pH 7.5, 150 mM NaCl, 0.1% Triton X-100, 0.1% Sodium deoxycholate, 1 mM EDTA, 1 mM Phenyl methyl sulphonyl fluoride (Sigma-Aldrich), Phosphatase inhibitor (PhosStop, Roche Applied Science), and protease inhibitor cocktail (Sigma-Aldrich). Nuclear proteins were isolated using NE-PER Nuclear and cytoplasmic extraction kit (Thermo Scientific). Protein concentrations were measured by Bradford’s protein assay kit (Bio-Rad Laboratories) using BSA as standard. Equal amounts of protein were separated on 8% SDS-PAGE at 100 V and transferred onto 0.4 µ thick nitrocellulose membrane for 1.5 hours at 200 mA. After blocking with 5% BSA or BLOTTO (Santa Cruz Biotechnology) prepared in Tris-buffered saline, Tween, 0.1%, (TBST) membranes were incubated with respective primary antibody diluted in blocking buffer for 16 hours at 4°C. Membranes were then washed in TBST for 10 minutes twice and incubated with horseradish peroxidase-conjugated secondary antibody. Primary and secondary antibodies used are listed in Table S1. The signals were detected using HyGLO Quick spray western blot detection reagent (Denville Scientific Inc., Metuchen, NJ). The band intensity was measured by Quantity One gel analysis software (Bio-Rad Laboratories).

### Liver Explant Culture

Embryonic livers from E12.5 embryos were collected, washed in DMEM and cultured on Whatman Nucleopore Track-Etch membranes (8 µm, Whatman Inc. Florham Park, NJ) for 3 days in a cell culture incubator at 37°C ± rFGF10 and ± ICG-001. Cultured livers were fixed in 4% PFA and paraffin blocks were prepared. 5 µm sections were stained for anti-phospho-Serine-552 β-CATENIN as described earlier.

### Statistical Analysis

ANOVA-Post hoc Fisher’s PLSD test was performed using Statview software (SAS Institute Inc., Cary, NC**)** to calculate statistical significance. *p*<0.05 was considered as significant.

## Results

### FGF Signaling in E12.5 Murine Hepatoblasts

**Figure 1:**
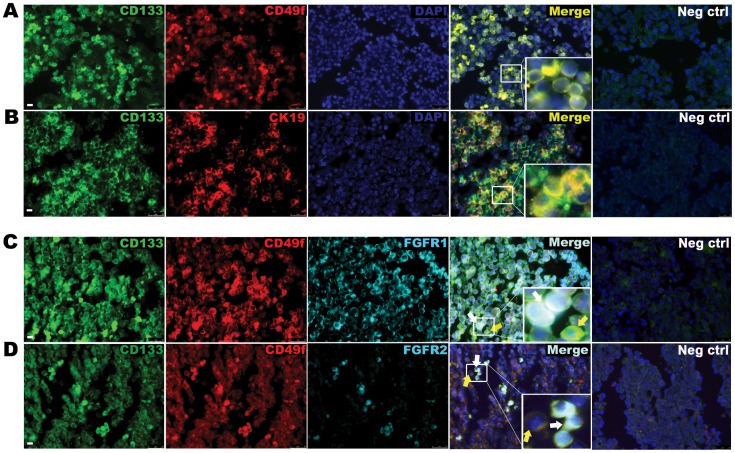
The murine E12.5 liver is populated with CD133^pos^CD49f^pos^ hepatoblast progenitor cells expressing FGFR1 and 2. (A) Immunofluorescence staining for progenitor cell markers CD133 and CD49f on E12.5 embryonic liver. Yellow cells are double positive cells. DAPI staining (blue) identifies nuclei. (B) Immunostaining of CD133 and Cytokeratin (CK19). (C) Immunostaining for CD133, CD49f and FGFR 1 and (D) FGFR2. White arrows mark triple positive cells and yellow arrows mark positive cells that do not express receptors. Data are representative of three or more independent experiments. Scale bar represents 25 µm.

We previously showed that E12.5 murine hepatoblasts, which exhibit FGF10-dependent β-catenin, also co-express *Albumin*, *Cytokeratin-19*, and *Fgfr2IIIb* [36]. Herein, we first sought to characterize histologically the hepatoblasts in E12.5 livers. Previous studies have demonstrated that hepatic progenitor cells express a variety of cell surface markers such as CD133 (also known as Prominin) and CD49f (also known as Integrin alpha-6) [4]–[9]. We observed co-expression of CD133 and CD49f (Figure 1A) as well as co-expression of CD133 and CK19 (Figure 1B). We calculated that approximate 35% of cells were CD133^pos^CD49f^pos^. Similarly, 35% of cells were CD133^pos^CK19^pos^. We, therefore, extrapolated that approximately 35% of total liver cells are CD133^pos^CD49f^pos^CD19^pos^. Notably, subsets of CD133^pos^CD49f^pos^ cells also co-expressed FGFR1 or FGFR2 (Figure 1C, 1D).

**Figure 2:**
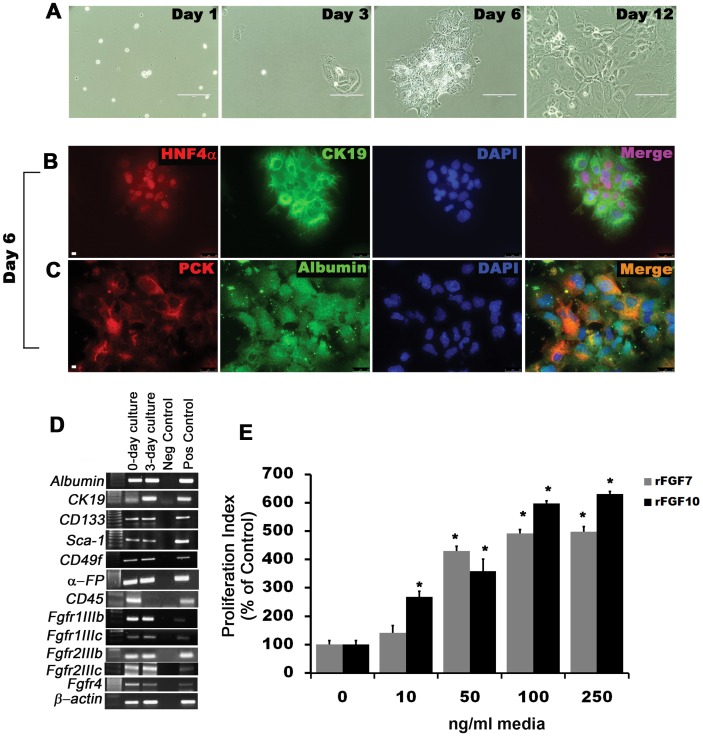
Isolation and *in vitro* expansion of CD133^pos^CD49f^pos^CD45^neg^ cells by Magnetic Assisted Cells Separation (MACS) from E12.5 liver enriches for hepatoblast progenitor cells that proliferate in response to FGFR activation. (A) Phase contrast pictures of *in vitro* expanded CD133^pos^ CD49f^pos^ CD45^neg^ enriched cells from E12.5 embryonic liver at different time points. Co-immunofluorescence staining for (B) HNF4α and CK19 and (C) ALBUMIN and PCK. (D) Gene expression analysis by RTPCR using RNA isolated from the MACS enriched cells before and after 3-day culture. Negative control = water and positive control = E16.5 whole embryonic cDNA. Scale bar 25 µm. (E) Proliferation indices of CD133^pos^ CD49f^pos^ CD45^neg^ cells treated with rFGF7/10 for 48 hrs and pulse labeled with BrdU (n = 4, *p<0.001 compared to control). Data are representative of three or more independent experiments.

To further evaluate the progenitor nature of embryonic CD133^pos^ CD49f^pos^cells, we enriched for these cells using Magnetic Assisted Cell Separation (MACS) methodology. We first depleted CD45^pos^ cells to remove hematopoietic cells from the suspension of total liver cells given that the embryonic liver is a major site for extra-medullary hematopoiesis during embryogenesis; CD45 depletion consistently resulted approximately in a 50% reduction in total liver cells. We then positively selected the remaining CD45 depleted cells for CD133 and CD49f. The final cell suspensions were then plated onto Laminin-coated culture dishes, where the cells grew in monolayered colonies of small epithelioid-type cells. Beyond one week, however, many cells began to lose their oval shape and high nuclear-to-cytoplasmic ratio as well as their colony forming nature while becoming more fibroblast-like morphologically (Figure 2A). RTPCR analysis of the three-day cultured cells demonstrated expression of putative stem cell markers *Cd133*, *Cd49f*, *Sca-1*, *α-fetoprotein* as well as co-expression of hepatocyte genes *Albumin* and *Hepatocyte nuclear factor-4α* (*Hnf4α*), biliary epithelial gene *Ck19*, and a number of *Fgf receptors*, particularly *Fgfr1IIIb* and *Fgfr2IIIb*, which are generally expressed by epithelial cells (Figure 2B). *Fgfr4*, which encodes a hepatocyte-specific receptor, was also expressed. Immunofluorescence-based co-localization of HNF4α and CK19 as well as pan-CYTOKERATIN (PCK) and ALBUMIN is consistent with bipotentiality of expanded MACS-enriched cells after 6 days in culture (Figure 2C, 2D). *Cd45* expression was present at day zero indicating some contamination of the plated cell population; however, *Cd45* was undetectable three days later possible due to attrition of hematopoietic cells. At both zero and three days of culture, we noted expression of *Fgfr1IIIc* and *Fgfr2IIIc*, which are generally expressed by mesenchymally-derived cells. However, even by six days of culture, there was no detectable expression of mesenchymal marker DESMIN (Figure S1). All experiments with primary putative hepatoblast culture were, hereafter, completed within 6 days of plating in order to maximize epithelial progenitor cell phenotype and minimize potential epithelial mesenchymal transition.

We then proceeded with the assumption that our primary embryonic liver cultures were sufficiently enriched with hepatoblasts for the purposes of this study. We further speculated that these epithelially derived cells likely express Fgfr1IIIb and Fgfr2IIIb, and, therefore, would proliferate in response to FGF7 or FGF10. We sought to determine the effect of dose escalation of rFGF7 and rFGF10 on primary hepatoblasts *in vitro*. We observed significant increases in proliferation index up to five-fold and six-fold with increasing concentrations of rFGF7 and rFGF10, respectively, from 0 to 250 ng/mL (Figure 2E, p<0.001). There was no evidence of inhibition across all doses with higher concentrations.

### FGF Signaling Activates Downstream AKT and ERK Pathways and Promotes Cellular Proliferation in Mat1a*^−/−^* Tumor Initiating Cells

**Figure 3:**
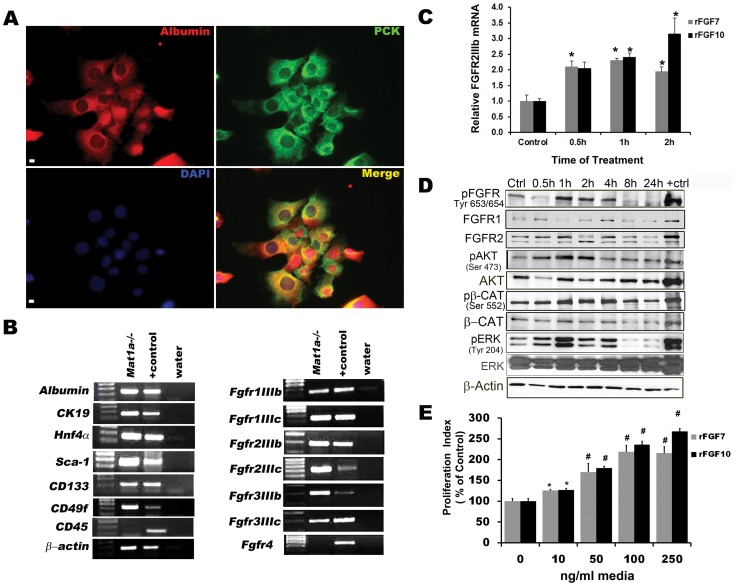
FGFR activation promotes downstream activation of AKT, ERK, and β-catenin pathways as well as proliferation of *Mat1a* *^−/−^* **cells.** (A) Co-localization of ALBUMIN and pCK in *Mat1a^−/−^* cells. Scale bar 25 µm. (B) RTPCR analysis shows *Mat1a^−/−^* cells express putative stem gene markers and *Fgfr*’s. Positive (+) control E16.5 = embryonic cDNA. Data are representative of three or more independent experiments. (C) qPCR analysis shows that rFGF7/10 upregulates expression of *Fgfr2IIIb* (n = 3, *p<0.01). (D) Western blot analysis of time course rFGF7 treatment on *Mat1a^−/−^* cells results in the downstream phosphorylation and activation of FGF receptor, AKT, ERK and β-CATENIN (ctrl (control) = untreated cells, +ctrl = E12.5 embryonic lung). Data are representative of two independent experiments. (E) Proliferation indices for *Mat1a^−/−^* cells treated with rFGF7/10 for 48 hrs and pulse labeled with BrdU (n = 4, *p<0.001 compared to control).

Given concerns of potential plasticity of our primary hepatoblasts in culture, we also chose to study FGF signaling activation in an established and relatively stable tumor initiating stem cell line derived from the livers of *Mat1a^−/−^* mice in order to elucidate differences and commonalities between the two cell culture models. Rountree *et al.*, previously expanded clones of single CD133-expressing non-parenchymal cells isolated by fluorescence-activated cell sorting (FACS) from *Mat1a^−/−^* livers. The authors showed that these cells express *Ck19*, *Albumin*, *Hnf4α*, *and α-Fetoprotein* in a manner consistent with bi-potential progenitor cells. Furthermore, these cells could be passaged multiple times *in vitro* and transplanted subcutaneously into nude mice where they developed into small tumors of clonally expanded hepatic epithelial stem cells [37]. To maintain our *Mat1a^−/−^* cells as phenotypically as a tumor initiating epithelial stem cell line, we further enriched for CD133 and CD49f by MACS. By immunofluorescence staining, we then demonstrated co-expression of ALBUMIN and pan-CYTOKERATIN (PCK), consistent with the previously established bipotential nature of these cells (Figure 3A). There was no observable expression of DESMIN (Figure S1C). Plated *Mat1a^−/−^* cells exhibited cuboidal, epithelial-like morphology in colonies. RTPCR gene expression analysis of *Mat1a^−/−^* cells revealed co-expression of hepatocyte specific genes (*Albumin* and *Hnf4α*), biliary specific gene (*Cytokeratin-19*), and stem cell/progenitor cell genes (*Sca-1*, *Cd133*, *Cd49f*, *α-Fetoprotein*). *Cd45* and *Fgfr4* were not detectable. *Mat1a^−/−^* cells expressed *IIIb* and *IIIc* isoforms of *Fgfr1*, *Fgfr2*, *and Fgfr3*, indicating potentially some degree of epithelial mesenchymal transition at the gene expression level (Figure 3B).

We then treated these cells with rFGF7 or rFGF10. In both cases by qPCR, we observed a greater than 2-fold increase in *Fgfr2IIIb* mRNA level after 30 minutes of stimulation (Figure 3C, n = 3, p<0.01). Stimulation with rFGF7 increased the phosphorylation of FGFR, which recognizes all FGFR (120–130 kDa) phosphorylated at Tyrosine 653/564 (Figure 3C). Given that both FGF7 and FGF10 have much greater affinity for FGFR2IIIb than any other FGFR including FGFR1IIIb [15], [16], it is likely that the observed phosphorylation and, therefore, activation is predominantly that of FGFR2IIIb. While total FGFR cannot be determined, there was no appreciable increase in either FGFR1 or FGFR2 even though there was evidence of increased *Fgfr2IIIb* expression. Downstream of FGFR activation by rFGF7, we observed phosphorylation/activation of AKT (Serine 473) and ERK1/2 (Tyrosine 204), two known downstream targets of activated FGFR signaling, as well as β-CATENIN (Serine 552, Figure 3D). Moreover, treatment with either rFGF for 48 hours resulted in a significant 2- to 2.5-fold increase in proliferation of *Mat1a^−/−^* HPCs in culture (Figure 3E, *p*<0.001 and *p*<0.05).

### FGF Signaling Induces AKT-dependent β-catenin Activation in Both Mat1a*^−/−^* Cells and Embryonic Hepatoblasts

**Figure 4:**
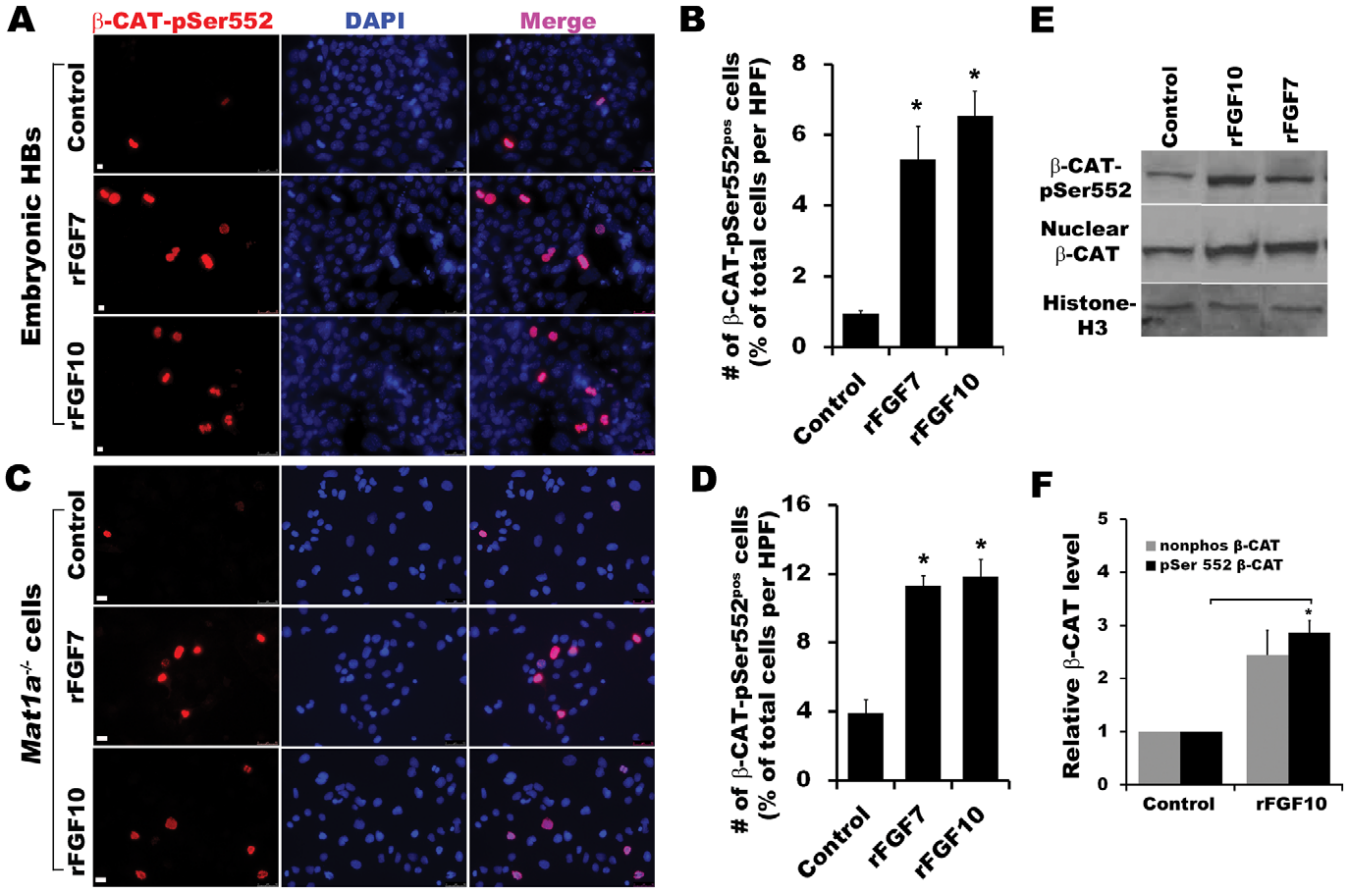
FGFR signaling increases the activation and nuclear localization pSer-552 β-catenin in hepatoblast and *Mat1a* *^−/−^* ***cells***. Immunofluorescence staining and quantification for pSer-552 β-CATENIN in rFGF7/10-treated MACS-enriched day-4 CD133^pos^ CD49f^pos^ CD45^neg^ (A, B) and *Mat1a^−/−^* cells (C, D). Total number of pSer-552 β-CATENIN positive cells were counted in 3–4 HPF from three independent experiments each and represented as % of total cells (n = 3, *p<0.001). Scale bar 25 µm. (E, F) Western blot analysis of nuclear extracts from *Mat1a^−/−^* cells treated with rFGF10. HISTONE-H3 was used to normalize for relative nuclear protein expression levels (n = 3, *p< 0.005).

**Figure 5:**
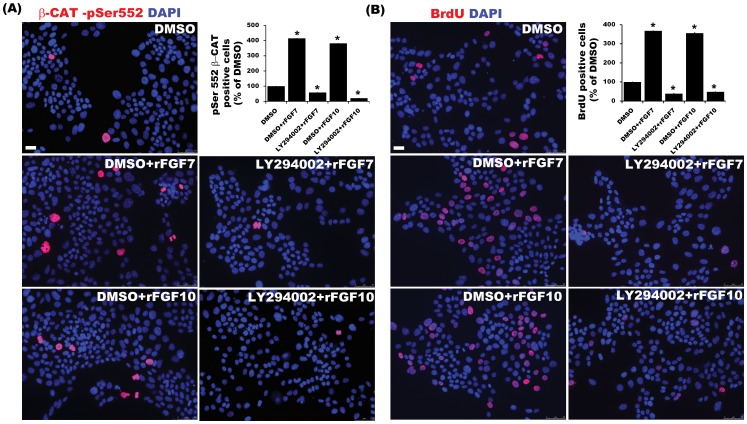
FGFR mediated proliferation of *Mat1a* *^−/−^* **cells are mediated in part through PI3K-AKT pathway.** Serum-starved *Mat1a^−/−^* cells were treated with rFGF7/10 ± PI3K-AKT inhibitor LY294002 for detection and quantification of (A) pSer 552 β-CATENIN or (B) BrdU. Total and β-CATENIN/BrdU Positive cells were counted from 4–5 HPF images from 3 independent experiments and represented as relative % of control (n = 3, *p<0.001).

FGF10 signaling induces downstream β-catenin activation leading to proliferation and enhanced survival of hepatoblasts during hepatogenesis [36]. Given the role of PI3K signaling in liver bud growth, [17] we sought to determine if PI3K activation might play a role in the activation of β-catenin in hepatoblasts and *Mat1a^−/−^* cells downstream of FGFR activation. Accordingly, we analyzed CD133^pos^CD49f^pos^ enriched hepatoblasts derived from E12.5 liver and *Mat1a^−/−^* cells histologically for nuclear β-CATENIN phosphorylated at Serine 552 (pSer-552), the site of AKT-dependent phosphorylation and transcriptional activation [24]. In both cases, media supplementation with either rFGF7 or rFGF10 resulted in 3- to 6-fold increases in the number of cells positive for nuclear pSer-552 β-CATENIN (Figure 4A–D, *p*<0.001, n = 3). Similarly, western blot analysis of the nuclear extracts from *Mat1a^−/−^* cells stimulated with rFGF10 demonstrated a ∼3-fold increase in pSer552-β-CATENIN (Figure 4E–F). The increase in pSer-552 β-CATENIN was not associated with an increase in total β-CATENIN (Figure 3D). Co-treatment of *Mat1a^−/−^* cells with rFGF7/10 and AKT inhibitor LY294002 resulted in complete abrogation of pSer-552 β-CATENIN up-regulation as well as BrdU incorporation (Figure 5A–B, p<0.001).

**Figure 6:**
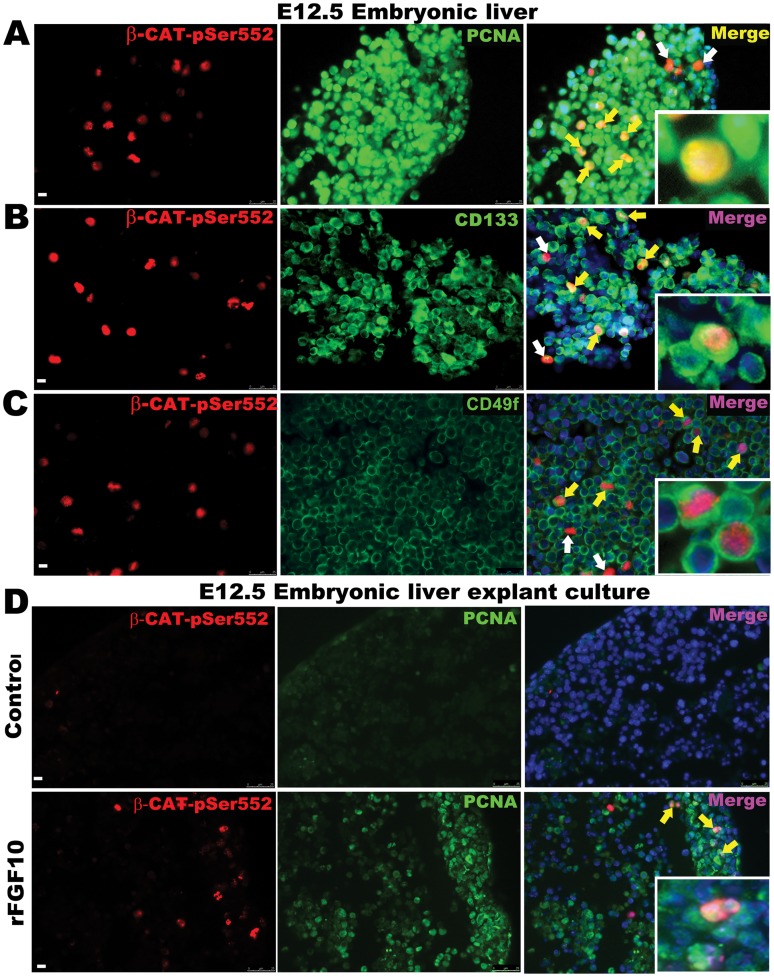
AKT-mediated activation of β-catenin in E12.5 embryonic liver. Co-staining for (A) pSer-552 β-CATENIN and PCNA, (B) pSer-552 β-CATENIN and CD133, (C) pSer-552 β-CATENIN and CD49f *in vivo*. (Di & ii) Co-staining for pSer-552 β-CATENIN and PCNA on E12.5 liver explant cultured for three days ± rFGF10. Data are representative of three or more independent experiments. White arrows represent single positive cells and yellow arrows show double positive cells. Scale bar = 25 µm.

*In vivo* immuno-histologic analysis of E12.5 livers showed that the majority of the pSer-552 β-CATENIN expressing cells are co-positive for PCNA, CD133, CD49f and E-CADHERIN but not DESMIN (Figure 6A–C and Figure S2B–C). Notably, however, not all proliferating cells are pSer-552 β-CATENIN positive indicating that other pathways are involved in parallel to promote proliferation. AKT-dependent activation of β-catenin is also observed in the proliferating cells at later stages (E18.5) of embryonic liver development (Figure S2A). Similarly, e*x vivo* E12.5 liver explants cultured in the presence of rFGF10 demonstrated increased levels of pSer-552 β-CATENIN and proliferation (Figure 6Di–ii). These observations indicate that FGF signaling promote proliferation of hepatoblasts partly through AKT-depended β-catenin activation.

### FGFR Activation Promotes β-catenin-dependent Cell Cycle Progression of Embryonic Hepatoblasts and Mat1a*^−/−^* Cells through a CBP Dependent Pathway

**Figure 7:**
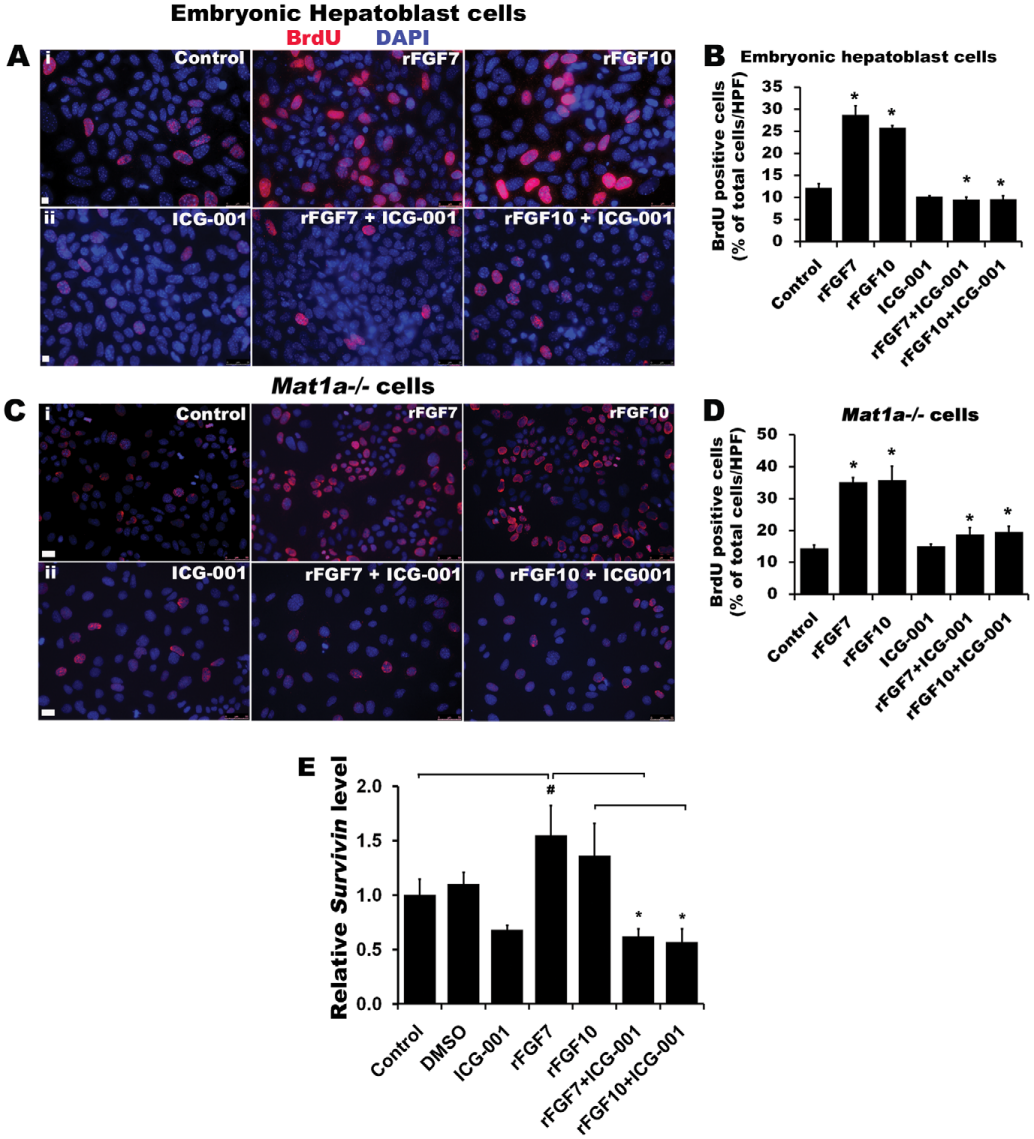
Activation of FGF signaling drives embryonic hepatoblasts and *Mat1a* *^−/−^* cells into the cell cycle through a CBP-dependent pathway. Immunodetection and quantification of BrdU positive cells in (A, B) CD133^pos^ CD49f^pos^ Cd45^neg^ enriched embryonic hepatoblasts (3-day cultured) and (C, D) *Mat1a^−/−^* cells after treating with rFGF7 or rFGF10 ± β-catenin-CBP inhibitor ICG-001. Total number of BrdU positive cells were counted from 3–4 HPF from three independent experiments and represented as % of total cells (n = 3, *p<0.0001 versus control). FGF-ICG-001 was compared to respective control FGF treated experiments. (E) qPCR analysis of *Survivin* mRNA expression level in *Mat1a^−/−^* cells for control, DMSO control, and rFGF7/10 ± ICG-001 groups. Expression levels are normalized to *Actin* (n = 3, *p< 0.005, #*p*<0.05).

To determine the role of the co-activator CBP in FGFR-mediated activation of β-catenin on cell cycle progression, BrdU incorporation assays were performed on CD133^pos^CD49f^pos^ enriched embryonic hepatoblasts and *Mat1a^−/−^* cells. FGFR activation by rFGF7 or rFGF10 for 3 hours resulted in nearly 3-fold increase in BrdU positive cells in both cell types (Figure 7A–D, n = 4, *p*<0.001). Co-treatment with ICG-001, an inhibitor of the CBP/catenin interaction [38], completely abrogated BrdU incorporation and, hence, initiation of cell proliferation induced by FGFR. FGFR activation by rFGF10 resulted in a 50% increase in the expression of *Survivin*, a downstream anti-apoptotic gene target of CBP-β-catenin co-activation [39], whereas ICG-001 co-treatment nearly completely blocked this effect (Figure 7E, *p*< 0.005 and *p*<0.05, respectively). We, therefore, conclude that FGFR-mediated activation of β-catenin-CBP pathway promotes the survival and proliferation of hepatoblasts and *Mat1a^−/−^* tumor initiating stem cells at least in part via an anti-apoptotic mechanism.

## Discussion

FGF signaling is required for normal hepatogenesis [1]–[3]. FGF1, FGF2, and FGF8, which are secreted by adjacent cardiac mesenchyme, induce liver bud formation from the foregut endoderm. Previous studies have shown that initiation of liver bud growth is dependent on AKT pathway activation [17]. We previously reported that FGF10, secreted by embryonic hepatic stellate cells, promotes the proliferation and survival of hepatoblasts via activation of β-catenin pathway after the initial liver bud formation [36]. In this study, we demonstrate that FGF7 and FGF10, likely through FGFR2IIIb, induce downstream β-catenin activation and its nuclear translocation in both post-liver bud embryonic hepatoblasts and *Mat1a^−/−^* tumor initiating stem cell line via AKT-dependent phosphorylation of Serine-552. We also show that FGF-induced proliferation of these hepatic progenitor/stem cells is mediated in part through the interaction of β-catenin with its co-activator CBP.

β-catenin activation is critical for normal hepatogenesis. Levels of both nuclear as well as total β-catenin increase during early liver development [19], [40]. We previously showed evidence that FGF10, secreted by embryonic hepatic stellate cells of the septum transversum, induces proliferation of hepatoblasts via β-catenin activation greatest around E12.5 [36]. In the current study, we show that FGF-dependent proliferation is mediated in part via AKT-dependent β-catenin activation in both hepatoblasts as well as in *Mat1a^−/–^*derived tumor initiating liver stem cells similar to that seen during liver bud formation [17]. He *et al* demonstrated that AKT-dependent activation of β-catenin promotes self-renewal of intestinal stem/progenitor cells [41]. Volckaert *et al* showed that FGF10 directly activates AKT and subsequently β-catenin via its phosphorylation at Ser-552 in lung progenitor cells to promote self-maintenance [42]. While our data do not exclude the role of Wnt-dependent canonical stabilization of β-catenin in progenitor cells through GSK-3β/Axin/APC complex, AKT-mediated FGF-dependent activation of β-catenin clearly contributes to hepatic progenitor/stem cell self-maintenance and renewal beyond the initial stages of hepatogenesis.

CBP/β-catenin interaction appears to be critical for the self-renewal of a variety of tissue stem cells, making this complex an intriguing therapeutic target for clinical disease. Emami *et al* previously showed that ICG-001 selectively binds the N-terminus of CBP, without binding to the highly homologous co-activator p300, leading to inhibition of proliferation and induction of apoptosis of a colon carcinoma cell line in part via decreased expression of *Cyclin D1* and *Survivin*, an anti-apoptotic gene [38]. Teo *et al* showed that ICG-001 corrects defects in neuronal differentiation associated with familial Alzheimer’s disease-associated Presenilin-1 mutations [35]. Henderson *et al* showed that administration of ICG-001 prevents and can reverse bleomycin-induced pulmonary fibrosis, in part via maintenance of the alveolar epithelium [43]. In this study, we show that disruption of the CBP-β-catenin interaction by ICG-001 at a previously established, cytostatic dose inhibits FGF-induced proliferation of embryonic hepatoblasts as well as *Mat1a^−/−^* cells. It is conceivable that FGF-induced β-catenin activation may regulate epithelial progenitors during fibrogenic liver injury.

β-catenin pathway activation is critical for normal hepatogenesis. β-catenin pathway activation is also present in numerous cancers including hepatoblastoma [11], [18], [44], [45]. Transcriptional interaction of β-catenin with its co-activators CBP and p300 must be tightly regulated during liver development to control the balance between proliferation and differentiation of stem/progenitor cells. Future studies will need to address the potential role that AKT-mediated phosphorylation of β-catenin at Ser552 plays in modulating CBP binding affinity. Taurin *et al* demonstrated in Cos7 cells that phosphorylation of Ser675, within the C-terminal region of β-catenin (amino acids 647–781) to which both CBP and p300 bind, promotes its interaction with CBP and enhances the proliferation of vascular smooth muscle cells [38], [46], [47]; the authors did not examine the effects on the p300/β-catenin interaction. Our findings demonstrate that β-catenin Ser552 phosphorylation regulates the proliferation and survival of hepatoblasts and liver tumor initiating stem cells in a CBP-dependent manner. Multiple kinase cascades can be integrated into the modulation of β-catenin co-activator interactions to control the critical decision between maintenance of potency and initiation of differentiation [46]. It is likely that the interaction of CBP-β-catenin versus p300-β-catenin is regulated via phosphorylation of multiple residues both within the co-activators and β-catenin thereby differentially regulating stem/progenitors versus differentiated cells. Since the interaction of β-catenin with CBP promotes proliferation and maintenance of potency, while its interaction with p300 promotes the initiation of differentiation of embryonic and cancer stem cells [31], [48], β-catenin may bind predominantly to CBP during early hepatogenesis to promote the proliferation of hepatoblasts, while during the later stages of hepatogenesis, it may preferentially partner with p300 to regulate hepatocyte or biliary differentiation of hepatoblasts.

In conclusion, we show that FGF pathway-induced proliferation of embryonic hepatoblasts and *Mat1a^−/−^* liver cancer stem cells is in part dependent on AKT-mediated β-catenin activation and subsequent CBP/β-catenin driven transcription. Our observation that embryonic hepatoblasts and *Mat1a^−/−^* cells appear to be regulated in a similar fashion suggests that the FGF-AKT- β-catenin/CBP pathway is mechanistically important and likely may be conserved in other conditions of liver injury or malignancy. Precise detailing of these pathways could lead to a better understanding of the role of transcriptional co-activator regulation of β-catenin activity and also facilitate the development of future therapeutics either in the realm of stem/progenitor cell-based therapies for liver diseases or chemotherapy for hepatic malignancies.

## Supporting Information

Figure S1 **Assessment of DESMIN staining in hepatoblasts and** ***Mat1a*** *^−/−^* **cells.** Immunofluorescence against DESMIN and DAPI (A) before and (B) after MACS. Notably DESMIN was not detectable after MACS. (C) DESMIN was not detectable in *Mat1a^−/−^* cells. Data are representative of three or more independent experiments. Scale bar 25 µm.
(TIF)

Figure S2 **Co-localization of pSer-552 β-CATENIN with** ***in vivo*** **embryonic liver.** Co-staining for (A) PCNA and pSer-552β-CATENIN in E18.5 liver. (B) Co-staining for pSer-552β-CATENIN and epithelial marker E-CADHERIN, (C) pSer-552β-CATENIN and mesenchymal marker DESMIN in E12.5 liver. White arrows mark single positive cells. Yellow arrows and inset depict co-positive cells. Data are representative of three or more independent experiments. Scale bar 25 µm.
(TIF)

Table S1 **Antibodies list.**
(DOCX)

Table S2 **Primer list.**
(DOCX)
