# Supplementary figures and images for: Fibroblast Growth Factor Receptor-Mediated Activation of AKT-β-Catenin-CBP Pathway Regulates Survival and Proliferation of Murine Hepatoblasts and Hepatic Tumor Initiating Stem Cells
Source: PLoS One. 2012 Nov 30;7(11):e50401. doi: 10.1371/journal.pone.0050401 (PMC3540100; doi:10.1371/journal.pone.0050401)

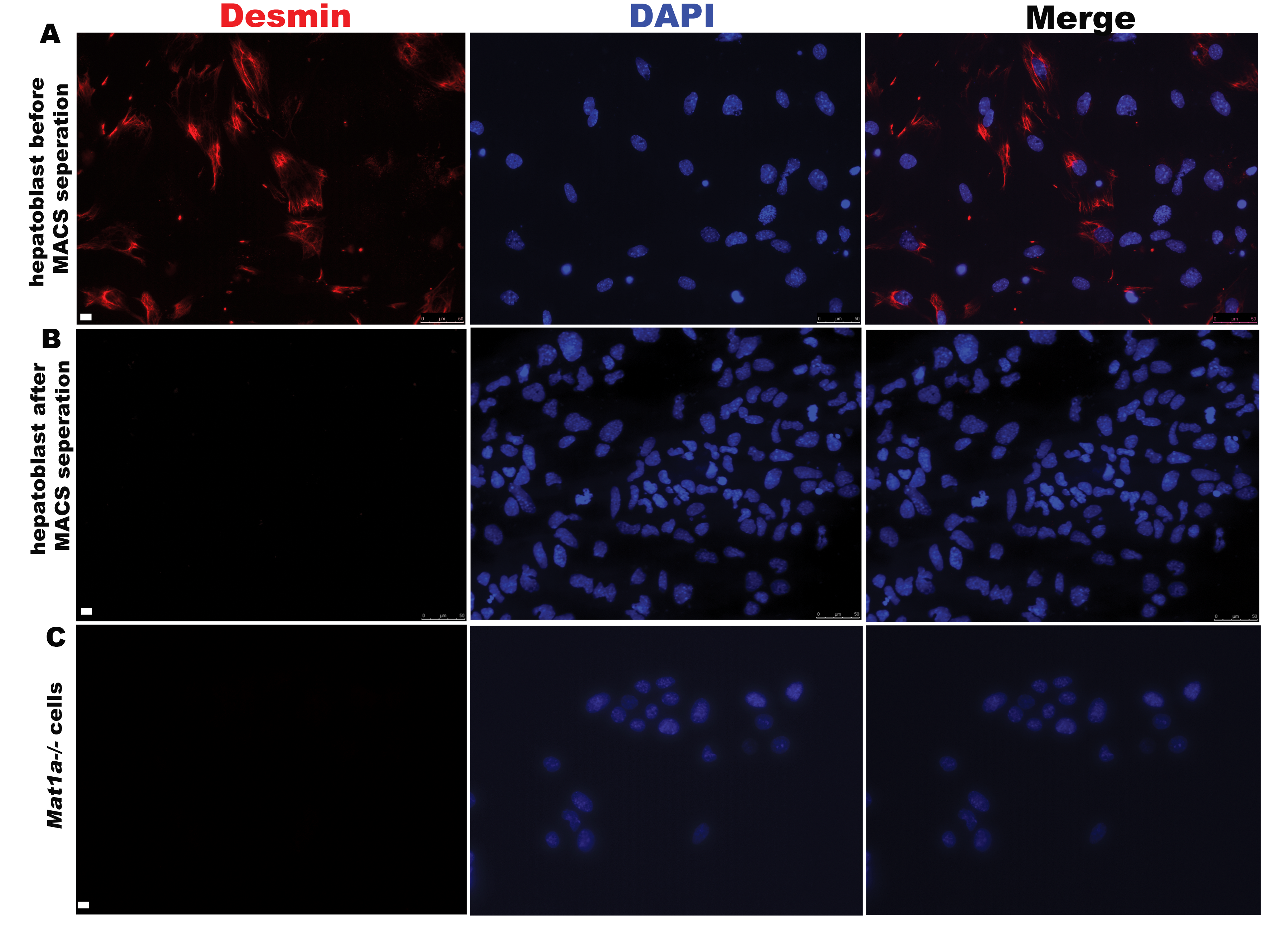

Supplement: Figure S1 — Assessment of DESMIN staining in hepatoblasts and Mat1a −/− cells. Immunofluorescence against DESMIN and DAPI (A) before and (B) after MACS. Notably DESMIN was not detectable after MACS. (C) DESMIN was not detectable in Mat1a−/− cells. Data are representative of three or more independent experiments. Scale bar 25 µm. (TIF) [file pone.0050401.s001.tif]

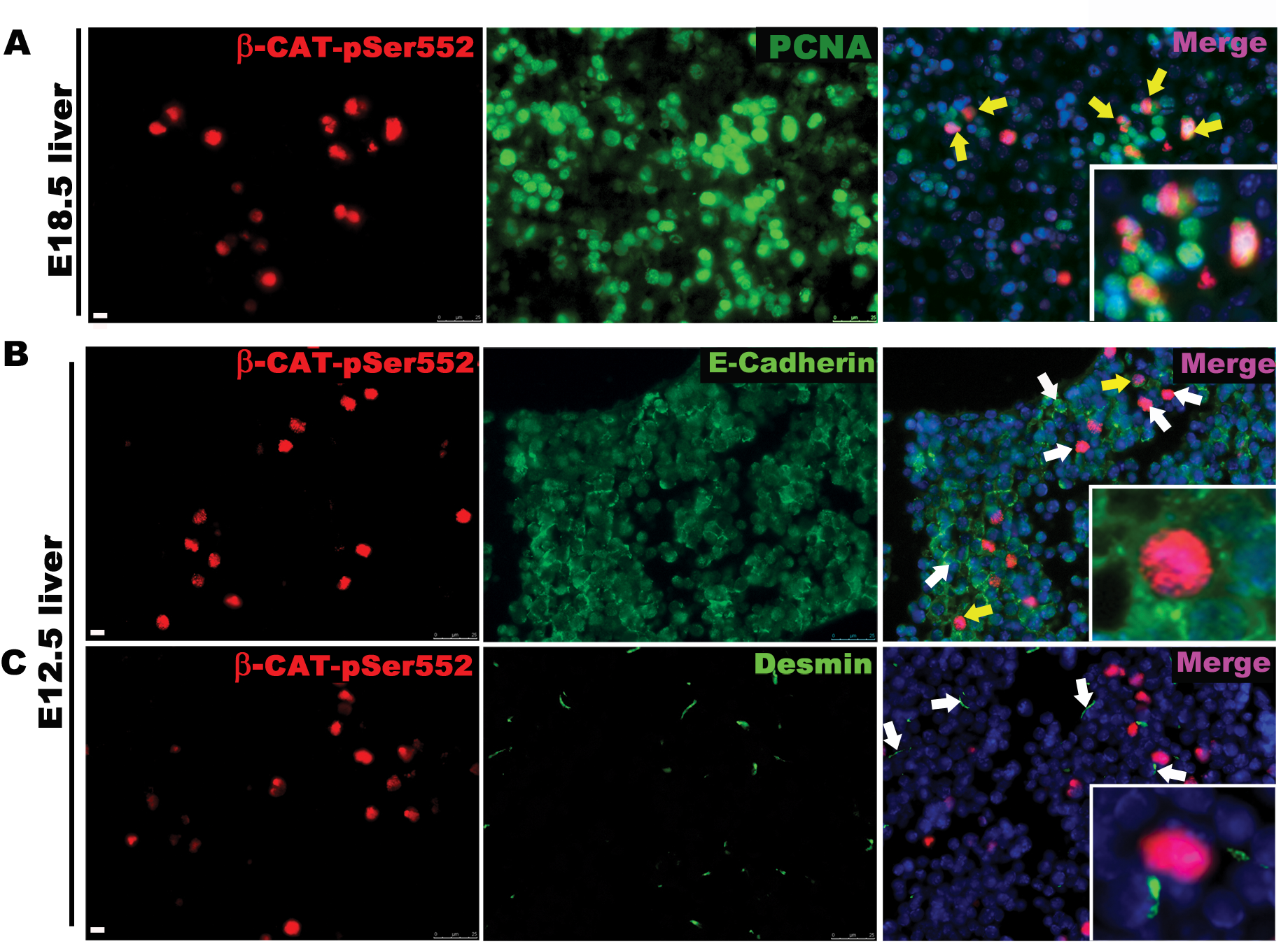

Supplement: Figure S2 — Co-localization of pSer-552 β-CATENIN with in vivo embryonic liver. Co-staining for (A) PCNA and pSer-552β-CATENIN in E18.5 liver. (B) Co-staining for pSer-552β-CATENIN and epithelial marker E-CADHERIN, (C) pSer-552β-CATENIN and mesenchymal marker DESMIN in E12.5 liver. White arrows mark single positive cells. Yellow arrows and inset depict co-positive cells. Data are representative of three or more independent experiments. Scale bar 25 µm. (TIF) [file pone.0050401.s002.tif]
