# Supplementary material for: Fibroblast Growth Factor Receptor-Mediated Activation of AKT-β-Catenin-CBP Pathway Regulates Survival and Proliferation of Murine Hepatoblasts and Hepatic Tumor Initiating Stem Cells
Source: PLoS One. 2012 Nov 30;7(11):e50401. doi: 10.1371/journal.pone.0050401 (PMC3540100; doi:10.1371/journal.pone.0050401)
Supplement: Table S1 — Antibodies list. (DOCX) [file pone.0050401.s003.docx]

**Table S1: Antibodies list**

| **Antibody** | **Company** | **Species** | **Dilution (IF)** | **Dilution**  **(western)** |
| --- | --- | --- | --- | --- |
| PCK | Sigma | Mouse | 1:100 |  |
| Albumin | Sigma | rabbit | 1:100 |  |
| HNF4α | Perseus proteomics | mouse | 1;100 |  |
| CD49f-PE | BD Pharmingen | rat | 1:50 |  |
| CD133 | eBioscience | mouse | 1:50 |  |
| Cytokeratin 19 | gift from Dr. Friedman | rabbit | 1:600 |  |
| BEK (FGFR2) | Abcam | rabbit | 1:100 |  |
| FLG (FGFR1) | Santa Cruz | rabbit | 1:100 |  |
| DESMIN | Dako | mouse | 1:100 |  |
| αSMA-Cy3 | Sigma | mouse | 1:100 |  |
| Vimentin | Dako | mouse | 1:100 |  |
| PCNA | Vector | mouse | 1:100 |  |
| pSer-552 β-catenin | gift from Dr. Li | rabbit | 1:100 | 1:1000 |
| Histone H3 | Cell Signaling | rabbit |  | 1:1000 |
| pFGFR | Cell Signaling | rabbit |  | 1:1000 |
| pAKT | Cell Signaling | rabbit |  | 1:1000 |
| AKT | Cell Signaling | rabbit |  | 1:1000 |
| pERK | Santa Cruz | mouse |  | 1:1000 |
| ERK | Santa Cruz | rabbit |  | 1:1000 |
| β-catenin | BD Transduction Lab | mouse |  | 1:1000 |
| β-actin | Sigma | mouse |  | 1:5000 |
