# Supplementary material for: Fibroblast Growth Factor Receptor-Mediated Activation of AKT-β-Catenin-CBP Pathway Regulates Survival and Proliferation of Murine Hepatoblasts and Hepatic Tumor Initiating Stem Cells
Source: PLoS One. 2012 Nov 30;7(11):e50401. doi: 10.1371/journal.pone.0050401 (PMC3540100; doi:10.1371/journal.pone.0050401)
Supplement: Table S2 — Primer list. (DOCX) [file pone.0050401.s004.docx]

**Table S2: primer list**

| **Gene** | **Forward** | **Reverse** |
| --- | --- | --- |
| **RTPCR primers primers** |  |  |
| ***Fgfr1b*** | 5′ tctggcctctacgcttgc3′ | 5′ aggatgggagtgcatctga3′ |
| ***Fgfr1c*** | 5′ tgctggagttaataccaccg3′ | 5′ ttccagaacggtcaaccatg3′ |
| ***Fgfr2b*** | 5′ ggcagtaaatacgggcctg3′ | 5′ ggacagtgagccaggcag3′ |
| ***Fgfr2c*** | 5′ aacgggaaggaggtttaagcag3′ | 5′ tggcagaactgtcaaccatgc3′ |
| ***Fgfr3b*** | 5′ gccaaccagacagccattctaggc3′ | 5′ cttggggcccgtgaacacgcagc3′ |
| ***Fgfr3c*** | 5′ cgatgcacagccacacat3′ | 5′ gttagcgcctgcagtcttg3′ |
| ***Fgfr4*** | 5′ gaatcgtattggaggcattcgg3′ | 5′ cagatacaagacctgtacctcc3′ |
| ***Cd133*** | 5′ accaacaccaagaacaaggc3′ | 5′ ggagctgacttgaattgagg3′ |
| ***Cd49f*** | 5′ gacggtgtttccctcaaaga3′ | 5′ tgcatcggaagtaagcctct3′ |
| ***Afp*** | 5′ tcaagaactcaccccaacct3′ | 5′ ggctctcctcgatgtgtttc3′ |
| ***Sca1*** | 5′ caggaggcagcagttattgtgg3′ | 5′ gggaggaccatcagagcaag3′ |
| ***Albumin*** | 5′ catgccaaattagtgcagga3′ | 5′ gctggggttgtcatctttgt3′ |
| ***Ck19*** | 5′ tgctggatgagctgactctg3′ | 5′ aatccacctccacactgacc3′ |
| ***Cd45*** | 5′ tggatttgcccttctggaca3′ | 5′ gtgtggattcagtggtgcga3′ |
| ***Hnf4a*** | 5′ actacggagcctcgagctgt3′ | 5′ agcccggaagcacttctta3′ |
| ***β-actin*** | 5′ tgacaggatgcagaaggaga3′ | 5′ cgctcaggaggagcaatg3′ |
| **qPCR primers** |  |  |
| ***Fgfr2IIIb*** | 5′cctacctcaaggtcctgaagc3′ | 5′catccatctccgtcacattg3′ |
| ***Survivin*** | 5′cccgatgacaacccgata3′ | 5′catctgcttcttgacagtgagg |
| ***β-actin*** | 5′ctaaggccaaccgtgaaaag3′ | 5′accagaggcatacagggaca3′ |
